# Does physical activity moderate the association between shorter leukocyte telomere length and incident coronary heart disease? Data from 54,180 UK Biobank participants

**DOI:** 10.1007/s11357-023-00890-7

**Published:** 2023-08-07

**Authors:** Meiruo Xiang, Luke C. Pilling, David Melzer, Ben Kirk, Gustavo Duque, Rui Liu, George A. Kuchel, Andrew R. Wood, Brad Metcalf, Breno S. Diniz, Melvyn Hillsdon, Chia-Ling Kuo

**Affiliations:** 1 Connecticut Convergence Institute for Translation in Regenerative Engineering, University of Connecticut Health Center, 195 Farmington Avenue, Suite 2080, Farmington, CT USA; 2 Epidemiology and Public Health Group, Faculty of Health and Life Sciences, University of Exeter, Exeter, UK; 3 Department of Medicine – Western Health, The University of Melbourne Australian Institute for Musculoskeletal Science (AIMSS), Saint Albans, Victoria, Australia; 4 Research Institute of the McGill University Health Centre, Montreal, Canada; 5 Department of Health Sciences, Sacred Heart University, Fairfield, CT USA; 6 UConn Center On Aging, University of Connecticut Health Center, Farmington, CT USA; 7 Genetics of Complex Traits, College of Medicine and Health, University of Exeter, Exeter, UK; 8 College of Life and Environmental Sciences, Sport and Health Sciences, University of Exeter, Exeter, UK; 9 Department of Public Health Sciences, University of Connecticut Health Center, Farmington, CT USA

**Keywords:** Accelerometer, Accelerometry, Moderation, Interaction, Population-based study, Prospective cohort study, Time to event survival data, Epidemiology

## Abstract

Telomere shortening is a biological aging hallmark. The effect of short telomere length may be targeted by increased physical activity to reduce the risk of multiple aging-related diseases, including coronary heart disease (CHD). The objective was to assess the moderation effect of accelerometer-based physical activity (aPA) on the association between shorter leukocyte telomere length (LTL) relatively in the population sample and incident CHD. Data were from the UK Biobank participants with well-calibrated accelerometer data for at least 6.5 days (*n* = 54,180). Relative mean LTL at baseline (5–6 years prior to aPA assessment) was measured in T/S ratio, using a multiplex quantitative polymerase chain reaction (qPCR) technology, by comparing the amount of the telomere amplification product (T) to that of a single-copy gene (S). aPA measures included total number of events (at least 10-s continued physical activity > 32 milligravities [mg]), total volume, mean duration, mean intensity, and peak intensity of all events. LTL, aPA measures, and their interactions were associated with incident CHD (mean follow-up 6.8 years) using Cox proportional hazards models adjusting for covariates. Longer LTL (relative to the sample distribution) was associated with reduced incidence of CHD (adjusted hazard ratio [aHR] = 0.94 per standard deviation [SD] increase in LTL, [95% CI, 0.90 to 0.99], *P* = .010). Incidence of CHD was reduced by higher total volume of aPA (aHR = 0.82 per SD increase in LTL, [95% CI, 0.71 to 0.95], *P* = .010) but increased by higher total number of events (aHR = 1.11 per SD increase in LTL, [95% CI, 1.02 to 1.21], *P* = .020) after controlling for other aPA measures and covariates. However, none of the interactions between LTL and aPA measures was statistically significant (*P* = .171).

**Supplementary Information:**

The online version contains supplementary material available at 10.1007/s11357-023-00890-7.

## Introduction

Telomeres are repetitive base pair sequences of TTAGGG at the end of chromosomes [1]. Critically short telomere length leads to permanent cell cycle arrest and cellular senescence [2]. Senescent cells secrete high levels of inflammatory cytokines, cell cycle regulators, growth factors, and tissue remodeling factors, known as the senescence-associated secretory phenotype (SASP) [3], which can exert deleterious effects on health outcomes, including cardiovascular disease [4]. Cellular senescence is one of the hallmarks of biological aging [5], and it can be a therapeutic target aiming to prevent or delay the onset of multiple diseases or conditions based on the geroscience hypothesis [6].

Whether physical activity is associated with telomere length remains inconclusive [7]. A systematic review and meta-analysis study [7] was conducted to evaluate the association between physical activity and telomere length. Most of the included studies measured telomere length in leukocytes using the quantitative polymerase chain reaction (qPCR) method. Different methods were used to measure physical activity, including questionnaires, physical activity monitors, and groups with different physical activity levels, e.g., athletes versus a control group. Partly due to the heterogeneity from measurements between studies, inconsistent findings on the association between physical activity and telomere length were reported: twenty studies did not find a statistically significant association, fifteen studies reported a positive association, while two studies described a U-shaped relationship.

It is unclear whether physical activity can be an intervention for telomere shortening [8]. Six randomized controlled trials were identified in the literature to examine the effect of physical activity on telomere shortening [8]. The meta-analysis results suggested that physical activity was not significantly associated with changes in telomere length. However, the estimates were uncertain as reflected in the wide confidence intervals. Furthermore, most of the included studies showed a moderate to high risk of bias from the randomization process or deviations from the protocol. Additionally, participants were predominantly obese or physically inactive so the findings may not be generalizable to the general population.

While it remains unclear if physical activity is associated with telomere length or telomere shortening that commonly occurs with aging, the benefits of physical activity for CHD is evident [9]. Being physically active reduces the incidence of CHD by 33–58% [9]. Results from a meta-analysis showed a dose–response relationship between physical activity and CHD [10]. The risk of CHD was reduced by 14%, comparing individuals who reported an equivalent of 150 min/week of moderate-intensity, leisure-time physical activity (LTPA) to those who reported no LTPA. More significantly, individuals who reported an equivalent of 300 min/week of moderate-intensity LTPA had a 20% lower risk of CHD than those who reported no LTPA. Similarly, the dose–response association was reported in a UK Biobank (UKB) study linking accelerometer measured physical activity to the incidence of cardiovascular disease [11].

The association of shorter LTL, relative to the sample distribution, with CHD is well documented in the literature [12] and the relationship is likely to be causal [13, 14]. Plausible mechanisms underlying the association between shorter LTL and CHD include oxidative stress [15], chronic inflammation [16], and endothelial cell senescence [17, 18], which may be reduced by physical activity [8, 19, 20]. We hypothesized that increased physical activity may mitigate the association between shorter LTL and CHD. To test the hypothesis, we conducted a prospective study using the UKB physical activity cohort with well-calibrated accelerometer data for at least 6.5 days (*n* = 54,180). This research is important as our findings will inform whether shorter telomere length can be targeted by physical activity to reduce the risk of incident CHD.

## Methods

### UK Biobank

Over 500,000 participants between ages 40 and 70 were recruited in the UKB between 2006 and 2010 [21]. Baseline assessments included detailed online questionnaires on health and lifestyle, physical measurements, and biological samples for future assays. Participants are followed up through linkages to electronic health records, including data from hospital admissions, primary care visits, cancer, and death registries. Subsets of participants returned to undergo additional assessments, e.g., multi-modality imaging scans and accelerometer-based physical activity (aPA) assessment [22].

### Study design

**Fig. 1:**
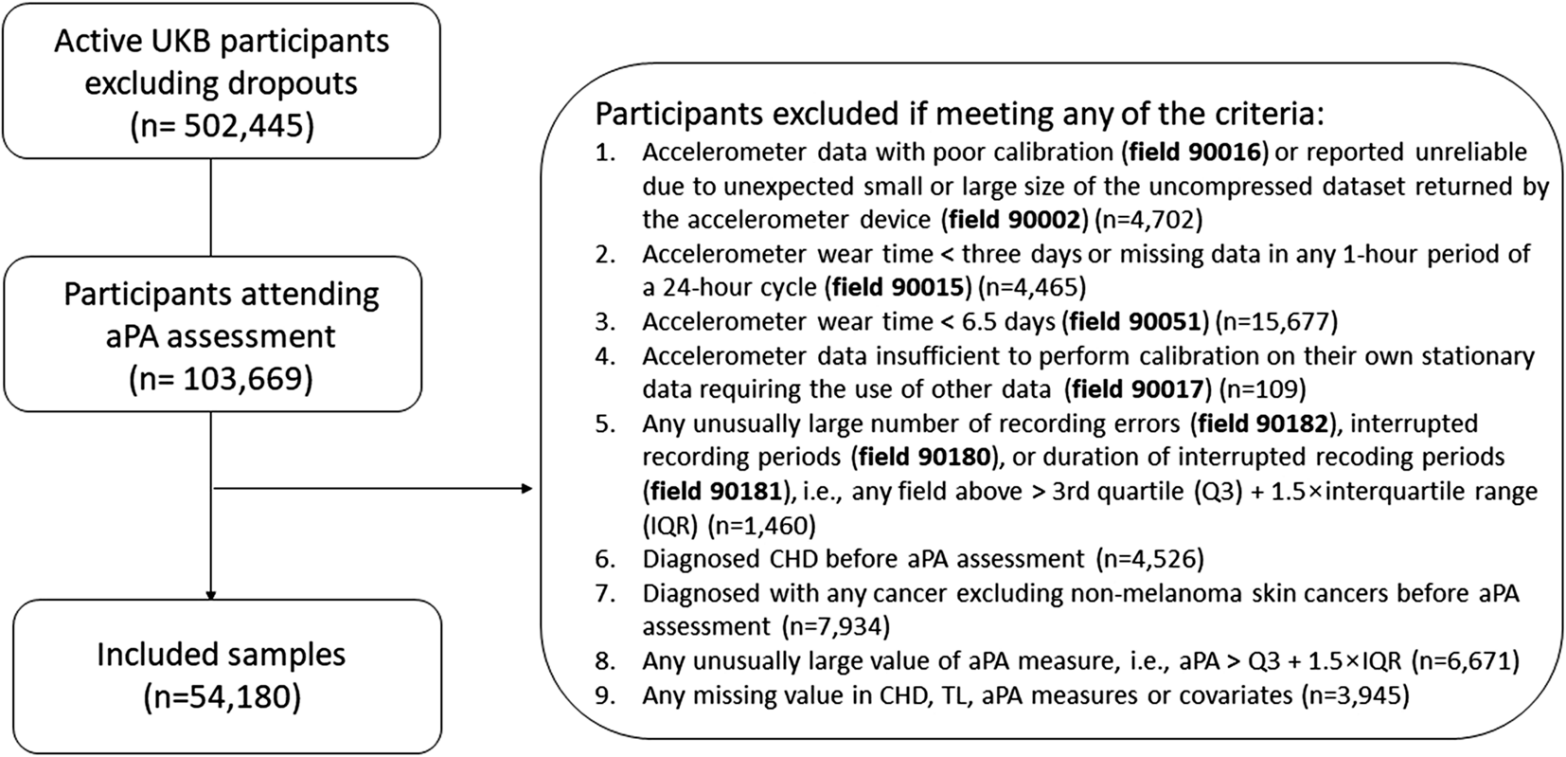
Study design: timeline of data collection

Baseline blood samples were used to measure LTL between 2006 and 2010. About 20% of the cohort participated in aPA assessment between 2013 and 2016. Since then, the included samples (detailed below) were followed up for incident coronary heart disease (CHD) via inpatient hospital episode statistics (HES) until death or the last censoring date depending on the country (9/30/2021 for England; 7/31/2021 for Scotland; 2/28/2018 for Wales) (Fig. 1).

### Included samples

**Fig. 2:**
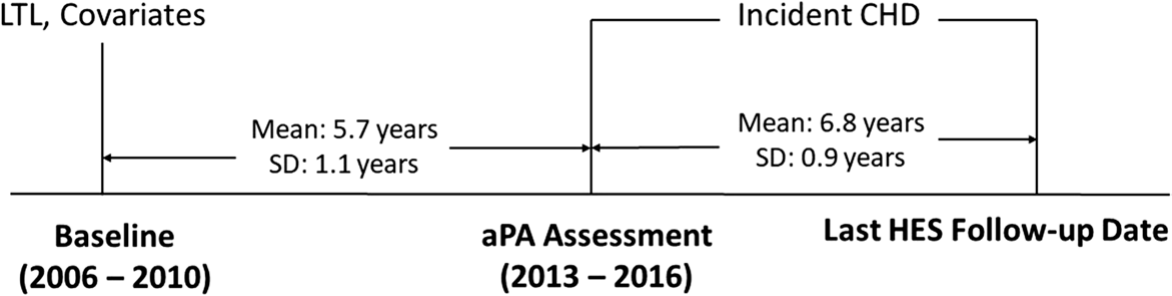
Study flow chart (last HES follow-up date: 9/30/2021 for England; 7/31/2021 for Scotland; 2/28/2018 for Wales)

Among 502,445 active UKB participants, regardless of ancestry, 103,669 completed the aPA assessment. We excluded participants who had accelerometer data with poor calibration or quality, or had short wear time (first five items in the exclusion box of Fig. 2). We also excluded (1) participants diagnosed with CHD or any cancer (excluding non-melanoma skin cancers) prior to aPA assessment, (2) participants with any unusually large value of aPA measure (i.e., greater than the third quartile + 1.5 × interquartile range), and (3) participants with any missing value in CHD, LTL, aPA measures, or covariates. As a result, a total of 54,180 participants were included in analysis.

### Data measurements

#### Leukocyte telomere length

DNA was extracted from peripheral blood leukocytes for all UKB participants at baseline (recruitment). Using a multiplex quantitative polymerase chain reaction (qPCR) technology, LTL referred to as the mean leukocyte telomere length was measured in T/S ratio, comparing the amount of the telomere amplification product (T) to that of a single-copy gene (S). LTL was adjusted for the influence of technical parameters by Codd et al. on behalf of the UKB [23]. Of those with LTL measurements at baseline in UKB (*n* = 488,400), 1884 participants had a second blood sample drawn between 2 and 10 years after the original sample, which was processed using the same methods and the LTL data were used to correct for regression dilution bias [23].

#### Disease diagnoses

Disease diagnoses (yes/no) were confirmed using baseline self-reported and inpatient HES data based on ICD-9 and ICD-10 codes. As described previously, we pre-excluded participants with any cancer (ICD-10 C00–C97; ICD-9 140–209), excluding non-melanoma skin cancers (ICD-10 C44; ICD-9 172). CHD cases were confirmed if any of the conditions were met: ICD-10 I20–I25; ICD-9 410–414. Time in years from aPA assessment to the first diagnosis of CHD was recorded for CHD cases and from aPA assessment to HES censoring date or date of death, whichever occurred first, for non-CHD controls.

#### Accelerometer-based physical activity

Participants attending the aPA assessment were asked to wear an Axivity AX3 triaxial accelerometer (https://axivity.com/product/ax3) on the wrist of their dominant hand, for 24 h a day for seven consecutive days [24]. There was no repeated aPA assessment at the time of manuscript submission. The acceleration was calibrated and summarized in x/y/z axes for each 1-s epoch using the Euclidean Norm Minus One (ENMO) [25]. We adopted an event-based approach to process aPA data. The event-based approach permits a robust analysis and quantification of patterns that facilitate physical activity research [26, 27]. A time series of “active” events was then extracted from the data. To identify the start and end of an “active” event, all values were capped at 40 milligravities (mg) to prevent skewing by outliers. The start of an event was defined as when the acceleration signal was equal to 40 mg and the end of the event was determined when the average acceleration for consecutive epochs fell below 32 mg (80% of 40 mg). This 80% criteria allowed for short periods when people may temporarily cease moving. Only events of at least 10 s were included in analysis to reduce the effect of wrist-specific/isolated movements on the data. Events were computed on a per-person and per-day basis. Each event was characterized by its start time, duration (seconds in each event), intensity (the mean value of acceleration in each event), and volume (the sum of accelerations in each event).

Five aPA measures were derived from the time series of events and the definitions are provided as follows.

1. **Total volume**: total volume (mg) of all active events
2. **Total number of events**: total number of active events
3. **Mean duration**: mean duration (seconds) of all active events
4. **Mean intensity**: mean of the mean intensity (mg/second) of all active events
5. **Peak intensity**: 95^th^ percentile of the 95^th^ percentile acceleration values (mg/second) within active events, which tends to increase with longer events

#### Baseline covariates

Demographic data included age, self-reported sex (male or female), ethnicity (grouped into White, Black, South Asian, and Other), and education (from none to college or university degree, see Table 1), as well as baseline assessment center near participant’s residence. Socioeconomic status was measured by the Townsend deprivation index, an area-based measure of material deprivation, with higher scores representing higher levels of deprivation. Lifestyle factors included smoking status, alcohol intake frequency, and body mass index (BMI). Smoking status (never, former, or current) and alcohol intake frequency (never, special occasions only, one to three times a month, one or twice a week, three or four times a week, daily or almost daily) were assessed at baseline via online questionnaires.

**Table 1:** Baseline characteristics of the included samples

Characteristics	Statistics^a^ (*N* = 54,180)
Age (years)	56.0 ± 7.7 (40, 70)
Follow-up years from baseline to aPA assessment	5.7 ± 1.1 (2.8, 8.6)
Follow-up years from aPA assessment to the last HES follow-up	6.8 ± 0.9 (2.2, 8.3)
Telomere length (T/S ratio), adjusted for the influence of technical parameters	0.8 ± 0.1 (0.2, 3.6)
Sex (= female)	30,894 (57%)
Ethnicity	
Black	420 (0.8%)
Other	678 (1.3%)
South Asian	474 (0.9%)
White	52,608 (97.1%)
Education	
None	4162 (8%)
CSEs or equivalent^b^	2012 (4%)
O levels/GCSEs or equivalent^c^	11,031 (20%)
A/AS levels/NVQ/HND/HNC^d^	10,075 (19%)
Other professional qualifications	2675 (5%)
College or university degree	24,225 (45%)
Townsend deprivation index	− 1.8 ± 2.8 (− 6.3, 10.6)
Body mass index (BMI)	26.7 ± 4.5 (14.1, 65.2)
Smoking status	
Never	31,690 (58%)
Previous	18,940 (35%)
Current	3550 (7%)
Alcohol frequency	
Never	2950 (5%)
Special occasions only	5036 (9%)
One to three times a month	5996 (11%)
Once or twice a week	13,597 (25%)
Three or four times a week	14,180 (26%)
Daily or almost daily	12,421 (23%)

^a^Statistics are mean ± standard deviation (minimum, maximum) for continuous variables and number of a particular level (% of *N*) for categorical variables

^b^*CSE*, certificate of secondary education

^c^*GCSE*, general certificate of secondary education

^d^*NVQ*, national vocational qualification; *HND*, higher national diploma; *HNC*, higher national certificate

#### Statistical methods

A descriptive analysis was conducted to summarize variables. The rank-based inverse normal transformation was applied to LTL and aPA measures among the included samples to normalize the data to zero mean and unit variance so the scales were unified before modeling. Following that, the Pearson correlation coefficients were calculated between aPA measures. The proportion of variance in total volume, attributed to other aPA measures, was estimated in a multivariable linear regression model.

Cox proportional hazards regression models were used to determine whether, and to what extent, LTL and aPA measures individually or all together were associated with incident CHD, adjusting for baseline covariates (age, sex, ethnicity, education, Townsend deprivation index, BMI, smoking status, alcohol intake frequency, and assessment center). In the survival analysis, time from aPA assessment to incident CHD was censored at the last HES follow-up date (9/30/2021 for England; 7/31/2021 for Scotland; 2/28/2018 for Wales) or date of death, whichever occurred first.

The non-linearity of LTL or aPA measures were evaluated using cubic penalized spline functions with 10 splines in the basis (default). In the joint analysis of LTL and all aPA measures, we evaluated the interactions between LTL and aPA measures in a forward manner, considering the significant aPA measures (*P* < 0.05) by the order of *P*-values in the Cox proportional hazards regression model with all aPA measures and covariates. *P*-values smaller than 5% were considered statistically significant. All the statistical analyses were performed in R 4.1.0.

## Results

Table 1 shows characteristics of the included samples (*n* = 54,180) at baseline when LTL was measured. These participants were predominantly White (97.1%), female (57%), and well-educated (45% with a college or university degree). The mean age was 56.0 years (SD = 7.7) at baseline, with a mean follow-up of 5.7 years (SD = 1.1) to aPA assessment and 6.8 years (SD = 0.9) from aPA assessment to last HES update.

Participants tended to have better-than-average socioeconomic status and healthy lifestyles, with the mean Townsend deprivation index − 1.75 versus the population average of 0. The mean BMI was 26.7 (SD = 4.5). Fifty-eight percent of the samples were never smokers. Ninety-five percent of them drank, including 23% drinking daily or almost daily. The mean LTL (T/S ratio) for the whole sample was 0.8 (SD = 0.1) after adjusting for the influence of technical parameters.

During follow-up, 1986 incident CHD cases were identified, with the mean age at diagnosis 68.8 years (SD = 7.7). Of the CHD cases identified, 1505 cases were diagnosed between the aPA assessment and the start of COVID-19 pandemic (2/1/2020). The remaining cases (*n* = 481) were diagnosed after the pandemic started. Comparing the two periods, the incidence was similar, 528 cases per 100,000 person-years [before] versus 549 cases per 100,000 person-years [after], suggesting a minimal bias in CHD diagnosis due to COVID-19.

A descriptive summary of aPA measures, along with histograms to illustrate their distributions, is provided in Supplemental Table 1 and Supplemental Fig. 1. The Pearson correlations (*r*) between aPA measures after z-transformations were calculated (Supplemental Fig. 2). A higher mean intensity (*r* = 0.57) or peak intensity (*r* = 0.49) and a longer mean duration (*r* = 0.79) were correlated with a higher total volume. In contrast, a higher total number of events was correlated with a lower total volume (*r* =  − 0.16). Eighty-seven percent of the variance in total volume was explained by other aPA measures (Supplemental Table 2). The multivariable linear regression analysis showed that all aPA measures were significantly and independently associated with total volume and, interestingly, that all associations were positive after controlling for other aPA measures (Supplemental Table 2). There was no significant collinearity based on variance inflation factors (VIF < 10) associated with each aPA measure (Supplemental Table 2).

### Association between LTL and incident CHD

A scatterplot of incidence of CHD versus LTL showed an inverse linear relationship (Supplemental Fig. 3). Using Cox proportional hazards regression models, we showed a reduction of 18% in the risk of CHD per SD increase in LTL (unadjusted HR = 0.82, [95% CI, 0.79 to 0.86], *P* < 0.001). After adjusting for covariates, the association remained statistically significant, with a reduction of 6% in the risk of CHD per SD increase in LTL (adjusted HR [aHR] = 0.94, [95% CI, 0.90 to 0.99], *P* = 0.010). “Per SD” hereafter is referred to as per standard deviation increase in LTL or an aPA measure. Additionally, we tested the non-linearity of LTL association with incident CHD in the adjusted Cox regression model and the result was not statistically significant (*P* = 0.131).

### Associations between and aPA measures and incident CHD

**Fig. 3:**
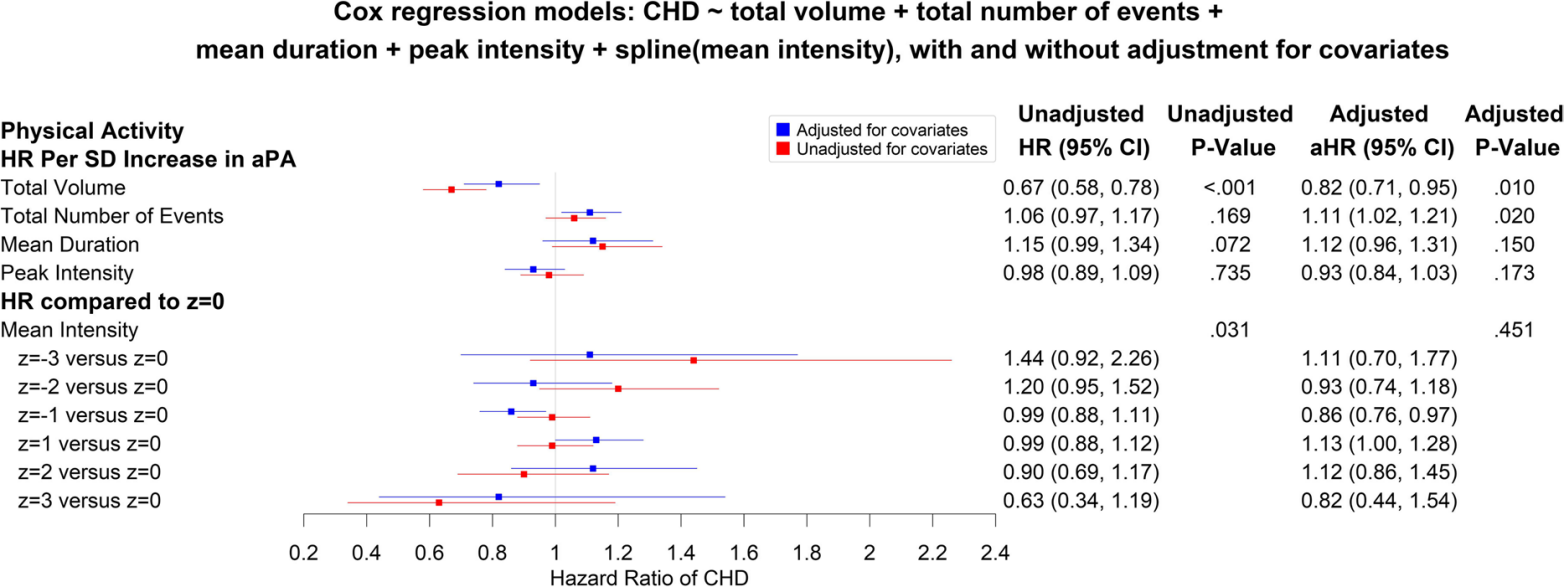
Associations between accelerometer-based physical activity (aPA) measures and incident coronary heart disease (CHD) risk in the Cox regression model for incident CHD on the aPA measures of total volume, total number of events, mean duration, and peak intensity, assuming linear relationships, and mean intensity via a cubic penalized spline function (non-linear function to account for significant non-linearity), without and with adjustment for the baseline covariates of age, sex, ethnicity, Townsend deprivation index, smoking status, alcohol intake frequency, and assessment center

The relationship between each aPA measure and incidence of CHD, as shown in Supplemental Fig. 4, was approximately linear. When they were modeled jointly in a multivariable Cox proportional hazards regression model, allowing for non-linear relationships with incident CHD, mean intensity was the only one showing significant non-linearity (*P* = 0.016). In the adjusted Cox regression model with a cubic penalized spline function (non-linear function) of mean intensity and other aPA measures assuming linear relationships, total volume and total number of events were the only two statistically significant aPA measures. For every 1-SD increase in total volume of aPA, the risk of incident CHD was reduced by 18% (aHR = 0.82 per SD, [95% CI, 0.71 to 0.95], *P* = 0.010), and the risk of incident CHD was increased by 11% per SD increase in the number of events (or aHR = 1.11 per SD, [95% CI, 1.02 to 1.21], *P* = 0.020) when other aPA measures and covariates are controlled. Of note, mean intensity modeled via a cubic penalized spline function was not significantly associated with incident CHD (*P* = 0.451) after adjusting for covariates (Fig. 3). The HRs comparing a mean intensity *z*-score to the mean of mean intensity *z*-scores (= 0) are selectively included in Fig. 3.

### Interactions between TL and aPA measures on incident CHD

For visualization, we categorized each aPA measure into low, medium, and high, corresponding to the quartile groups 0–25% (low), 25–75% (moderate), and 75–100% (high). As shown in Supplemental Fig. 5, longer LTL was associated with reduced incidence of CHD, and the three aPA groups showed similar slopes.

In the association analysis between aPA measures (including a non-linear function of mean intensity) and incident CHD, total volume (*P* = 0.010) and total number of events (*P* = 0.020) were the only two aPA measures that reached the significance level of 5% after adjusting for covariates and other aPA measures. Given the main effects of LTL and all aPA measures plus covariates, we first included the interaction term between LTL and total volume and the interaction *P*-value was not statistically significant (standardized *β* = 0.011, [95% CI − 0.033 to 0.055], *P* = 0.626) so the interaction term was not included in the model. Next, we included the interaction term between LTL and total number of events and the interaction term was not statistically significant either (standardized *β* = 0.005, [95% CI, − 0.039 to 0.049], *P* = 0.831). We also ran an overall test for any significant interactions between LTL and aPA measures by comparing the model with the main effects of LTL, all aPA measures, and covariates versus that with additional terms for interactions between LTL and individual aPA measures. Consistently, the result was not statistically significant (*P* = 0.171), which indicated that none of aPA measures significantly modified the risk of CHD associated with shorter LTL.

### Independent associations of LTL and aPA measures with incident CHD

**Fig. 4:**
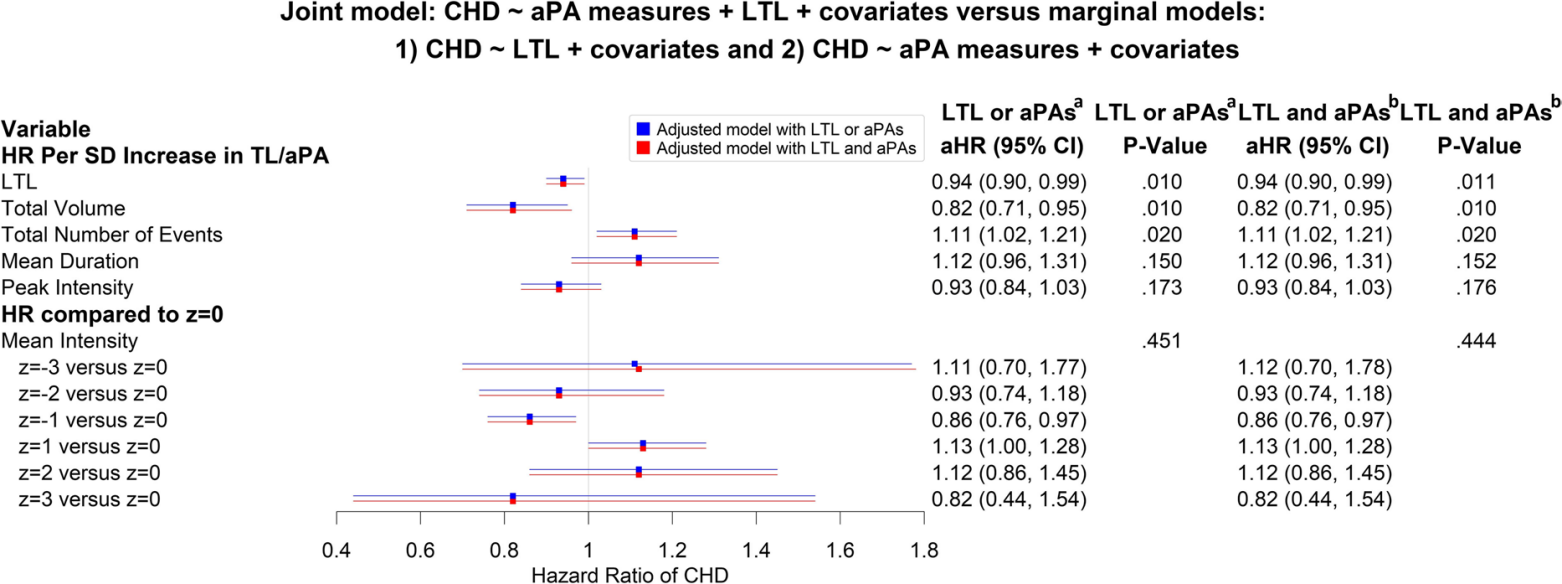
Joint model of leukocyte telomere length (LTL) and accelerometer-based physical activity (aPA) measures (total volume, total number of events, mean duration, and peak intensity assuming linear relationships and mean intensity via a cubic penalized spline function to account for significant non-linearity) versus models with LTL or aPA measures only. ^a^**LTL only**: CHD ~ LTL + age + sex + ethnicity + education + Townsend deprivation index + smoking status + alcohol intake frequency + baseline assessment center; **aPAs only**: CHD ~ aPA measures (total volume, total number of events, mean duration, and peak intensity assuming linear relationships and mean intensity via a cubic penalized spline function to account for significant non-linearity) + age + sex + ethnicity + education + Townsend deprivation index + smoking status + alcohol intake frequency + baseline assessment center. ^b^**LTL and aPAs**: CHD ~ LTL + aPA measures (total volume, total number of events, mean duration, and peak intensity assuming linear relationships and mean intensity via a cubic penalized spline function to account for significant non-linearity) + age + sex + ethnicity + education + Townsend deprivation index + smoking status + alcohol intake frequency + baseline assessment center

There was a lack of evidence that aPA measures moderate the association between LTL and incident CHD. LTL and aPA measures were independently associated with incidence of CHD, which was suggested by minimally changed aHRs associated with LTL and aPA measures (absolute differences smaller than 0.01), comparing the model with both LTL and aPA measures and the models with either LTL or aPA measures (Fig. 4). Additionally, total volume of aPA was most strongly associated with incident CHD after adjusting for other aPA measures. We also did not find a significant association between LTL and total volume (dependent variable) after adjusting for other aPA and covariates measures (standardized *β* associated with LTL 0.0008, [95% CI, − 0.0022 to 0.0038], *P* = 0.600) or covariates only (standardized *β* associated with LTL 0.0015, [95% CI, − 0.0065 to 0.0095], *P* = 0.710).

## Discussion

To our knowledge, this is the first large population-based study to evaluate the interaction of LTL with aPA measures on the risk of incident CHD. Longer telomere length was significantly associated with reduced risk of CHD. Likewise, higher total volume and lower total number of events of aPA were also significantly associated with reduced incidence of CHD. However, none of aPA measures showed a significant moderation effect on shorter LTL and incident CHD. Our results also suggested that LTL and aPA measures are independently associated with incident CHD.

The non-significant finding on the moderation of aPA measures is unlikely due to inadequate statistical power, as the 95% confidence interval for the standardized *β* associated with the interaction between LTL and an aPA measure is extremely short and centered around the null value zero. For example, when considering the interaction between LTL and total volume in the model with the main effects of LTL, aPA measures, and covariates, the standardized *β* and 95% confidence interval for the interaction was 0.011 ([95% CI, − 0.033 to 0.055], *P* = 0.626), and that when considering the interaction between LTL and total number of events was 0.005 ([95% CI, − 0.039 to 0.049], *P* = 0.831).

We have shown that higher total volume and lower number of events of aPA were significantly associated with decreased risk of CHD when modeled simultaneously with other aPA measures and adjusting for covariates (Fig. 3). For any given higher total volume, with fewer events, either the mean duration or the mean intensity must be higher compared to the same volume with more events. While it might be expected that lower mean intensity would be associated with a higher risk of CHD, in this multivariable model, it was associated with a lower risk of CHD for the following reasons. To achieve a higher total volume of aPA with the same number of events, a lower mean intensity must be accompanied by a longer mean duration and the duration offers the protection. By the same token, to achieve a higher total volume of aPA with the same number of events, a shorter mean duration must co-exist with a higher mean intensity, explaining why higher mean duration, in the multivariable model, appears to reduce the risk of CHD—albeit the associations with mean intensity and mean duration were not statistically significant (Fig. 3).

While several mechanisms have been postulated [28], a widely accepted hypothesis is that shorter telomeres lead to CHD through major SASP factors in different senescent cell types that contribute to atherosclerosis, e.g., IL-6 (forming atherosclerotic plagues and causing thrombosis), TNF (recruiting more immune cells and forming atherosclerotic plaques), and VEGF (promoting plaque angiogenesis and vascular remodeling) in senescent endothelial cells [29]. Physical activity may lower protein levels of SASP factors, which reduces the risk of CHD. While we found that none of the aPA measures significantly moderated the association of shorter LTL with incident CHD, a preclinical study showed that exercise decreased the protein expression of p53 and p16 (senescence markers) in endothelial cells from aortic lysates in young mice [17]. In a human study, the protein expression of p53, p21, and p16 (senescence markers) in endothelial cells from antecubital veins were significantly higher in older sedentary adults than in young sedentary adults, but the age-related differences were not present between older exercising adults and young sedentary adults [20]. Additionally, a recent study [30] showed that a 12-week structured exercise program significantly lowered the levels of several senescence-related proteins in peripheral blood CD3 + T cells, including p16, p21, cGAS, TNFα, and PD1, which may subsequently reduce the risk of incident CHD. Whether the improvement is universal or only applied to certain senescent cell types and tissues requires further investigation. A comprehensive study that accounts for the heterogeneity in major SASP factors in different senescent cell types and tissues is warranted to understand the relationships among short telomere length, physical activity, and senescence to CHD.

Several limitations need to be considered when interpreting the results. *First*, telomeres from cardiac tissues may be more relevant to this study than peripheral blood leukocytes, but the correlations between telomere length among different tissues are generally positive [31]. *Second*, LTL was only measured once at recruitment; therefore, we could not relate changes in LTL due to physical activity to the risk of CHD. *Third*, due to the unprecedentedly large sample size, the qPCR method was chosen for feasibility reasons but introduced higher variability than other techniques. To overcome the challenge, Codd et al. [23], on behalf of the UKB, removed the technical differences using statistical methods and the adjusted measurements warranted high intra-assay reliability and reproducibility based on the quality control evaluation. *Fourth*, although multiple measures are desired to assess different domains of physical activity, the 7-day aPA measures remain limited in modeling the temporal profiles of acceleration. *Fifth*, in addition to physical activity, other potential moderators such as demographics (e.g., age, sex, and ethnicity), lifestyle factors (e.g., smoking and alcohol intake), and disease status were not considered and the study samples of this present study were dominated by participants of European descent, with the mean age 56.0 years at aPA assessment. Likely, the moderation effect of physical activity may exist in subpopulations, which will require future investigation. *Sixth*, the study period includes the COVID-19 pandemic, which likely changed healthcare-seeking behaviors, but we found a similar incidence of CHD before and after the pandemic. *Lastly*, UKB participants are healthier than the general population, which may reduce exposure-outcome associations, but the effect is minimal due to significant heterogeneity in exposures [32].

In conclusion, longer duration physical activity events with a desired total volume are most beneficial for CHD, regardless of intensity. Physical activity, however, does not play a moderating role in the association between shorter LTL and incident CHD. Additional interventions are needed to reduce the adverse effects of shorter LTL on the risk of CHD.

### Supplementary Information

Below is the link to the electronic supplementary material.

Supplementary file1 (DOCX 3940 KB)

## Data Availability

Data used in this project are available via application to UK Biobank.
