# Supplementary material for: Does physical activity moderate the association between shorter leukocyte telomere length and incident coronary heart disease? Data from 54,180 UK Biobank participants
Source: GeroScience. 2023 Aug 7;46(1):1331–42. doi: 10.1007/s11357-023-00890-7 (PMC10828302; doi:10.1007/s11357-023-00890-7)
Supplement: Supplementary file 1 — Supplementary file1 (DOCX 3940 KB) [file 11357_2023_890_MOESM1_ESM.docx]

**Supplementary Information**

**Does physical activity moderate the association between shorter leukocyte telomere length and incident coronary heart disease?**

- **Data from 54,180 UK Biobank participants**

**Supplemental Table 1** | Summary statistics of accelerometer-based physical activity measures

**Supplemental Table 2** | Multiple linear regression model for total volume of accelerometer-based physical activity (aPA) with other aPA measures

**Supplemental Figure 1** | Distributions of accelerometer-based physical activity measures

**Supplemental Figure 2** | Pearson correlations between accelerometer-based physical activity measures after z-transformations

**Supplemental Figure 3** | Relationship between leukocyte telomere length and coronary heart disease incidence

**Supplemental Figure 4** | Relationships between accelerometer-based physical activity measures and coronary heart disease incidence

**Supplemental Figure 5** | Illustration of no interaction between leukocyte telomere length and each accelerometer-based physical activity measure on incident coronary heart disease

**Supplemental Table 1. Summary statistics of accelerometer-based physical activity measures**

|  | Minimum | First quartile | Median | Mean | Third quartile | Maximum | Standard deviation |
| --- | --- | --- | --- | --- | --- | --- | --- |
| Total number of events | 298.00 | 1102.00 | 1297.00 | 1321.13 | 1517.25 | 2162.00 | 295.50 |
| Mean duration (seconds/event) | 18.74 | 105.62 | 136.91 | 145.74 | 178.06 | 309.26 | 53.69 |
| Total volume (mg) | 518062.97 | 10762230.00 | 13732220.00 | 14031850.00 | 17033800.00 | 28210980.00 | 4604425.45 |
| Mean intensity (mg/second) | 43.59 | 60.14 | 64.28 | 64.64 | 68.79 | 83.52 | 6.21 |
| Peak intensity (mg/second) | 124.77 | 268.90 | 302.46 | 306.50 | 340.17 | 462.16 | 52.53 |

**Supplemental Table 2. Multiple linear regression model for total volume of accelerometer-based physical activity (aPA) with other aPA measures**

| **Independent variable:**  **aPA measure** | **Standardized *β* associated with each aPA measure (95% CI)** | ***P*-value** | **VIF^a^** | **Adjusted R-square** |
| --- | --- | --- | --- | --- |
| Total number of events | 0.519 (0.515, 0.523) | *P* < 0.001 | 1.74 | 0.87 |
| Mean duration | 1.032 (1.028, 1.036) | *P* < 0.001 | 2.00 |  |
| Mean intensity | 0.136 (0.129, 0.143) | *P* < 0.001 | 5.30 |  |
| Peak intensity | 0.135 (0.128, 0.142) | *P* < 0.001 | 4.91 |  |

^a^VIF: variance inflation factor


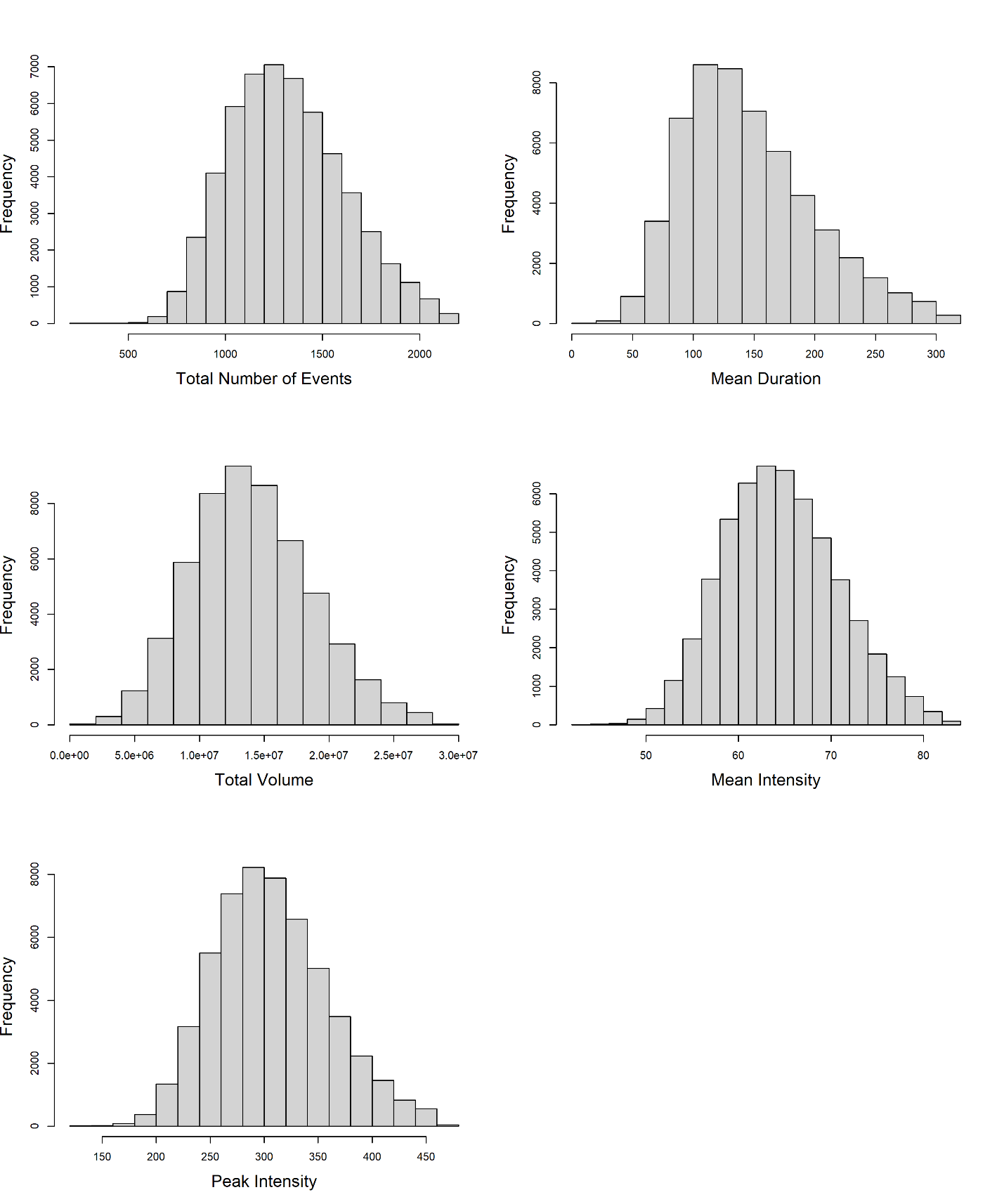


**Supplemental Figure 1. Distributions of accelerometer-based physical activity measures**

**
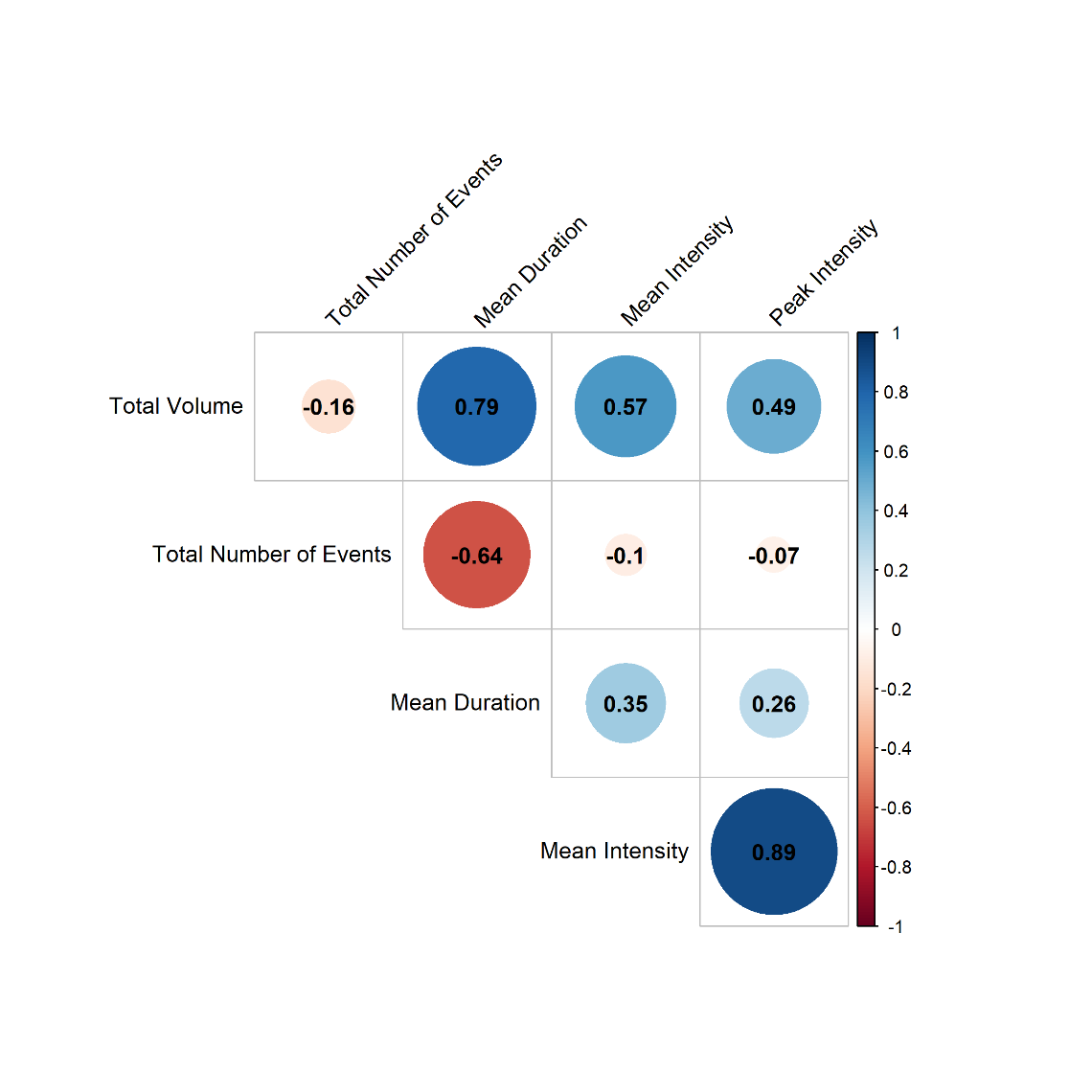
**

**Supplemental Figure 2. Pearson correlations between accelerometer-based physical activity measures after z-transformations**

**
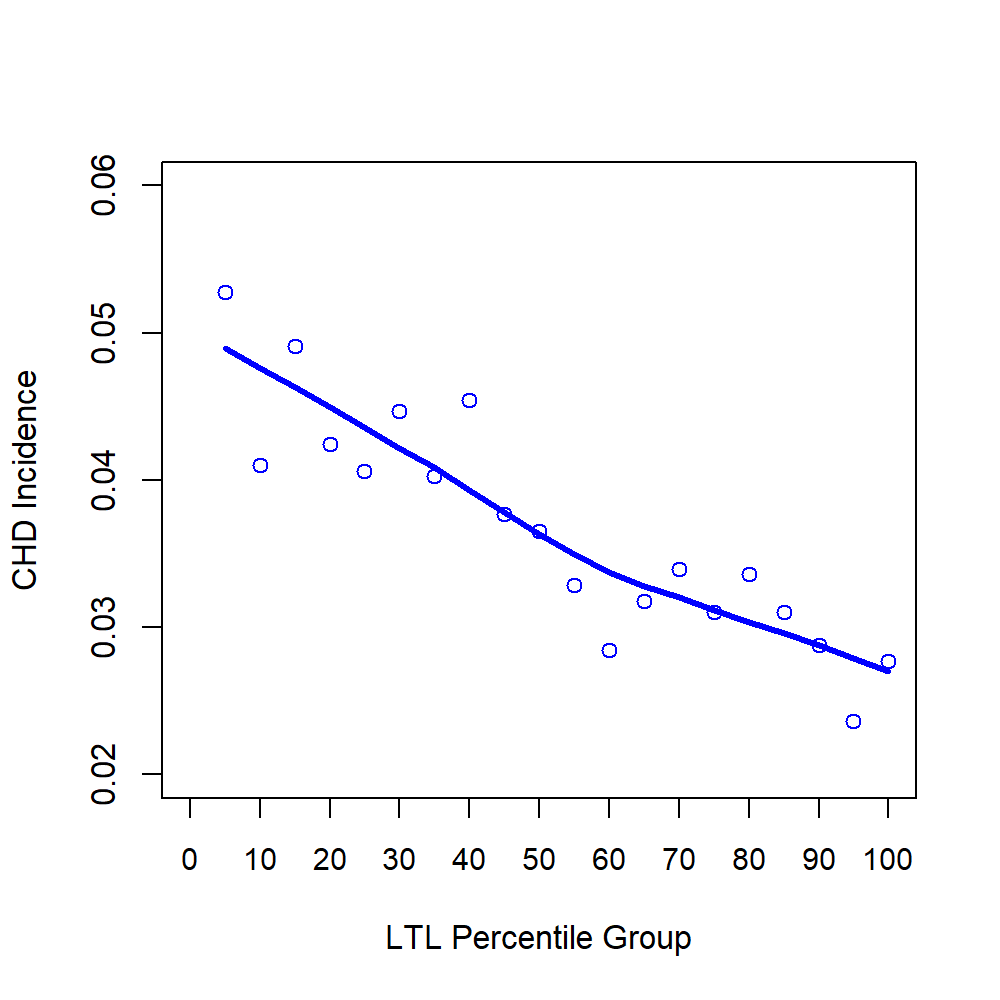
**

**Supplemental Figure 3. Relationship between leukocyte telomere length and coronary heart disease incidence**

Coronary heart disease (CHD) incidence versus leukocyte telomere length (LTL) percentile groups (0^th^-5^th^ percentile, 5^th^-10^th^ percentile, …., 95^th^-100^th^ percentile), including a LOWESS smooth curve using locally weighted polynomial regression (smoother span: 2/3 of points in the plot which influence the smooth at each value, with a larger value giving more smoothness).

**
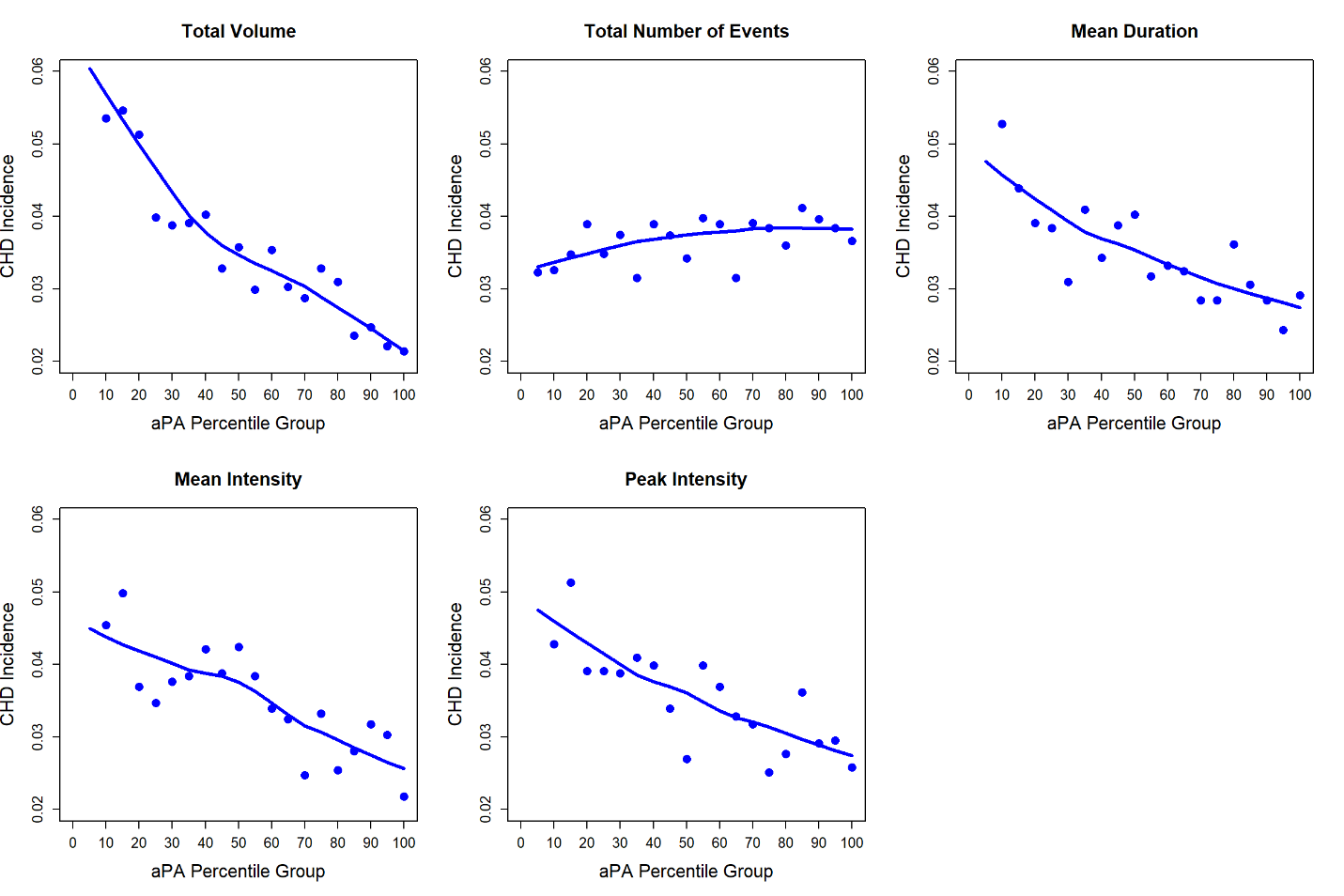
**

**Supplemental Figure 4. Relationships between accelerometer-based physical activity measures and coronary heart disease incidence**

Coronary heart disease (CHD) incidence versus accelerometer-based physical activity (aPA) percentile groups (0^th^-5^th^ percentile, 5^th^-10^th^ percentile, …., 95^th^-100^th^ percentile), including a LOWESS smooth curve using locally weighted polynomial regression (smoother span: 2/3 of points in the plot which influence the smooth at each value, with a larger value giving more smoothness).

**
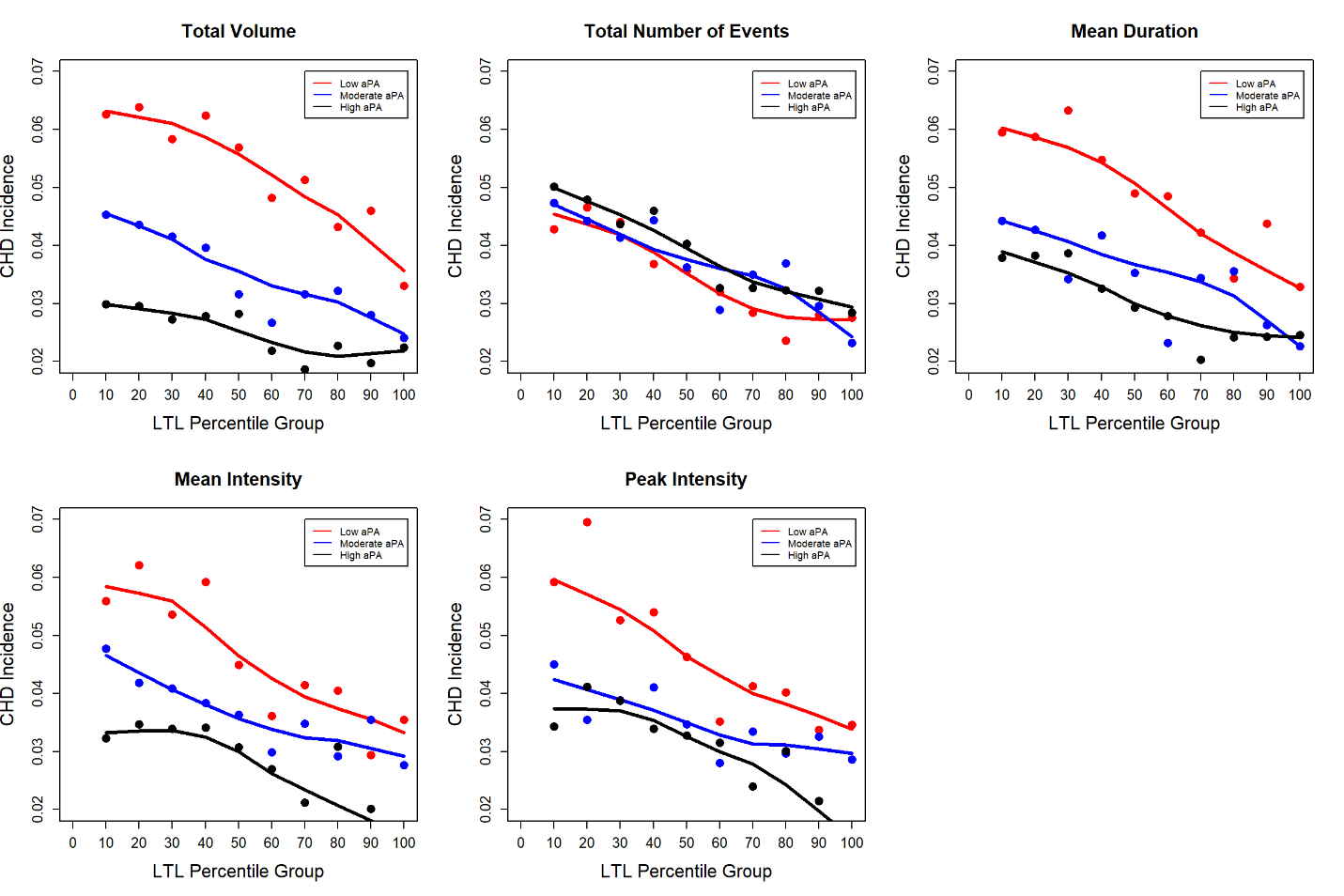
**

**Supplemental Figure 5. Illustration of no interaction between leukocyte telomere length and each accelerometer-based physical activity measure on incident coronary heart disease**

Coronary heart disease (CHD) incidence versus leukocyte telomere length (LTL) percentile groups (0^th^-10^th^ percentile, 10^th^-20^th^ percentile, …., 90^th^-100^th^ percentile) for low (0-25%), moderate (25-75%), and high (75-100%) groups of an accelerometer-based physical activity (aPA) measure, including LOWESS smooth curves based on locally weighted polynomial regression models (smoother span: 2/3 of points in the plot which influence the smooth at each value, with a larger value giving more smoothness)
